# Least Significant Change (LSC) for Serum Concentrations of 25-Hydroxyvitamin D

**DOI:** 10.3390/nu17132246

**Published:** 2025-07-07

**Authors:** Pawel Pludowski, Marek Wójcik, Maciej Jaworski, Agnieszka Ochocińska, William B. Grant, Michael F. Holick

**Affiliations:** 1 Department of Clinical Biochemistry, The Children’s Memorial Health Institute, 04-730 Warsaw, Poland; m.wojcik@ipczd.pl (M.W.); m.jaworski@ipczd.pl (M.J.); a.ochocinska@ipczd.pl (A.O.); 2 Sunlight, Nutrition, and Health Research Center, 1745 Pacific Ave., Ste. 504, San Francisco, CA 94109, USA; wbgrant@infionline.net; 3 Section Endocrinology, Diabetes, Nutrition and Weight Management, Department of Medicine, School of Medicine, Boston University, Boston, MA 02118, USA; mfholick@bu.edu

**Keywords:** 25-hydroxyvitamin D, least significant change, chemiluminescence, vitamin D, IDS-iSYS

## Abstract

**Background:** The least significant change (LSC) method should be introduced and considered a proper method to define the smallest clinically important difference between two consecutive measurements. **Methods:** The LSC was calculated based on 150 patients, with a total 25-hydroxyvitamin D [25(OH)D] IDS-iSYS assay performed in triplicate. The LSC was determined by multiplying the calculated root mean square precision error by a factor of 2.77. The study group was additionally divided into subgroups according to gender, age, serum 25(OH)D concentration, and date of assays. **Results:** The LSC was 4.0 ng/mL (13.2%) for the entire group (n = 150; 450 assays) and was not dependent on gender, age of patients, or the date of assays (*p* > 0.05). The LSC value depended only on the 25(OH)D concentration value. In the subgroup with vitamin D deficiency (<20 ng/mL), the obtained LSC value was 2.2 ng/mL (14.7%), which was lower compared to all other groups (*p* < 0.05 for insufficiency, and *p* < 0.0001 for the optimal concentration value). In the subgroup with 25(OH)D concentrations >50 ng/mL (n = 4; 12 assays), the calculated LSC was 11.8 ng/mL (16.9%) and differed statistically only from the subgroup with vitamin D deficiency (*p* < 0.005). **Conclusions:** An absolute LSC of 4.0 ng/mL was calculated for the IDS-iSYS assay used in our study and should be considered when two (or more) assay results of 25(OH)D performed for a single patient are compared.

## 1. Introduction

Modern medicine often uses statistical calculations; therefore, it seems reasonable to look for ways to make the best use of the obtained numerical data in practice [1,2,3,4,5].

The first definition used to assess the treatment process was the minimal clinically important difference (MCID), which is generally based on the assessment of the patient’s health condition and treatment progress [6]. The purpose of determining the MCID was to answer the question of how large the change should be to be clinically significant. Thus, the minimal clinically important difference was assumed to be the smallest change in treatment outcome that the patient considers subjectively perceptible. However, at the stages of medical procedures where there are only numerical data to evaluate (e.g., measurements of body composition or laboratory diagnostic results), it is necessary to apply a concept that considers only the obtained data.

The main parameter used to assess the received numerical results is precision, presented by means of standard deviation (SD) and the coefficient of variation (CV). Precision is defined as the degree of similarity of the results of multiple measurements using a single sample and a specific method; hence, it is a measure of repeatability [1,2,3,4,5]. Accuracy, in turn, is the degree to which a measurement matches the true value [1,2,3,4,5]. The objective of an analytical laboratory is to produce the best possible results in terms of both accuracy and precision. The quality of the results of a given laboratory is confirmed by appropriate validation procedures [3,5].

The main goal of evaluating a single test result is to confirm its consistency with the actual state, i.e., the real concentration of a given substance in circulation. Modern quality control systems are used for that purpose. The reference point is the concentration value measured by a reference method or calculated, e.g., as an average of all measurements.

The clinician making the diagnosis has a considerable number of laboratory and imaging tests at his disposal, as well as data from medical interviews, inter alia. All the information must be comprehensively analyzed based on the physician’s knowledge and experience. When comparing two or more results of the same biochemical parameter, e.g., blood vitamin D marker concentrations from different periods, the person interpreting the results may come across some doubts [7,8]. For example, whether two similar, “on the face of it”, 25(OH)D total concentration results obtained several months apart indicate that the patient is not supplementing cholecalciferol appropriately [7,8]. Or, does the correctness of the supplementation contribute to some laboratory error? Similar doubts may arise in any medical process where a result in a numerical form is interpreted.

Thus, it proved important to find and develop a suitable statistical concept allowing for a reliable assessment of whether the interpreted numerical results reflected the actual change that occurred in the patient.

Such a practical tool could be the least significant change (LSC). This value is defined as the smallest difference between consecutive measurements that can be considered a real and non-random change. The LSC is determined based on the previously calculated root mean square precision error [9,10,11,12].

The usefulness of the least significant change (LSC), a concept already known in theoretical statistics, was verified based on bone density and body composition measurements in a group of our pediatric hospital patients (at the age of 5–18) with the application of the dual-energy X-ray absorptiometry (DXA) method [10]. The study consisted of measuring bone mineral density (BMD) and body composition in a single DXA assay cycle with repositioning between measurements [10]. The primary objective of determining the LSC based on the DXA method in a pediatric patient population was to assess whether the change in the measured value observed during successive measurements was a real change and not a measurement error. Further, the same method was applied to calculate the LSC for peripheral quantitative computed tomography (pQCT) in our department [11,12]. A general rule of interpretation was adopted; if the difference in BMD or other variables assessed by DXA or pQCT in two consecutive measurements exceeded the established LSC value, it could be concluded with a 95% probability that the change was real [10,11,12].

As can be seen, the concept of the least significant change can be a useful tool for interpreting test results with a numerical value. Laboratory diagnostics is a discipline that mainly uses numerical data. To date, there have been no studies in scientific literature attempting to use LSC assessment to interpret 25-hydroxyvitamin D assay results. This justified the need to undertake this study, which presents a method of calculating the LSC for practical application.

The least significant change, presented as an absolute and relative value, was developed based on a representative sample of 150 patients for whom a routine 25(OH)D concentration assay was ordered. The Department of Clinical Biochemistry of the Children’s Memorial Health Institute performs tests for “25-hydroxyvitamin D total”, i.e., the sum of 25-hydroxyvitamin D2 from ergocalciferol (vitamin D2) and 25-hydroxyvitamin D3 from cholecalciferol (vitamin D3), both forms produced in the liver. The concentration of “25(OH)D total” is a marker of the body’s vitamin D status [7,8,13]. On behalf of the Departments and Clinics in the Children’s Memorial Health Institute, approximately 1500 samples are usually tested monthly to determine 25(OH)D, using the chemiluminescent method. The large number of tests performed by the Department of Clinical Biochemistry [13,14] allowed for the selection of an appropriate number of serum samples for practical determination of the LSC value. The test design was based on the similarity to the concept verified in the densitometric tests [9,10,11,12] and consisted of performing three rounds of 25(OH)D total determination in one analyzer cycle.

## 2. Materials and Methods

### 2.1. Study Group

The study was conducted in parallel with the routine determination of 25(OH)D total at the Department of Clinical Biochemistry, the Children’s Memorial Health Institute. A total of 150 standard serum samples were collected from outpatients, patients in wards, and those undergoing commercial testing. The age range of the study subjects (67 females and 83 males) was 1 month to 71 years old. The mean age in the study group was 11.8 years ± 10.9 years, and the majority of patients were in the growth and maturation period of life.

The study group (n = 150) was divided according to the following criteria: gender, age, 25(OH)D concentration, and the date of registration (blood draw) at the Children’s Memorial Health Institute. According to age, the patients were divided into 5 subgroups: under 1 year of age (n = 17), over 1 year of age to 3 years (n = 15), over 3 to 12 years of age (n = 43), over 12 to 18 years of age (n = 64) and over 18 years of age (n = 11). According to the 25(OH)D concentration, the following 4 reference ranges were investigated: 25(OH)D < 20 ng/mL (n = 33), 25(OH)D above 20 ng/mL to 30 ng/mL (n = 53), 25(OH)D above 30 ng/mL to 50 ng/mL (n = 60), and 25(OH)D above 50 ng/mL to 100 ng/mL (n = 4). Using the criterion of date of registration/date of blood collection, the study group (n = 150) was divided into 5 equal subgroups of 30 study subjects each. The separate subgroups covered the following time periods: group I, 27 December 2023 to 14 February 2024 (Winter set); group II, 14 February 2024 to 11 April 2024 (late Winter–Spring set); group III, 11 April 2024 to 7 June 2024 (Spring set); group IV, 8 June 2024 to 14 August 2024 (late Spring–Summer set); and group V, 19 August 2024 to 4 October 2024 (late Summer–Fall set).

### 2.2. Methods

In the period from the end of December 2023 to early October 2024, the 25(OH)D concentration values for a total group of 150 patients were determined. Single blood draws were performed on the patients at the blood collection center by qualified nursing staff. The total 25(OH)D concentration was determined routinely at the Department of Clinical Biochemistry using the IDS-iSYS diagnostic system and a method based on chemiluminescence technology (IDS 25 VitD^s^). The intra-assay repeatability declared by the IDS-iSYS manufacturer was 8.3% CV, on average, while the inter-assay repeatability was 12.1% CV. The practical measurement range (linearity) was from 4 ng/mL to 110 ng/mL, according to the IDS-iSYS system document provided for testing IDS 25 VitD^s^ from the manufacturer.

The study samples were selected randomly, with the only logistic criterion being enough serum to perform three parallel determinations. Each patient’s sample was distributed into 3 tubes, for which the 25(OH)D concentration was measured in a single run of the IDS-iSYS device (intra-assay repeatability).

Specifically, 10 μL of patient sample was subjected to a pre-treatment step to denature the vitamin D-binding protein (VDBP). The treated samples were neutralized in an assay buffer, and a specific anti−25(OH)D antibody labeled with biotin was added. Following an incubation step, acridinium-labeled 25(OH)D was added. Following a further incubation step, the magnetic particles linked to streptavidin were added. After the final incubation step, the complex was captured using a magnet, and a wash step was performed to remove any unbound analyte. Trigger reagents were added, and the resulting light emitted by the acridinium label was inversely proportional to the concentration of 25(OH)D in the original sample.

Statistical analysis was performed using Statistica software v.10 (StatSoft Inc., Tulsa, OK, USA). The normality of the distribution of variables was verified using the Shapiro–Wilk test. The Mann-Whitney test was used to determine the statistical significance of differences between the two subgroups. In the case of more than two subgroups, the non-parametric Kruskal–Wallis ANOVA test was used. When the overall ANOVA coefficient was less than 0.05, post hoc tests for multiple comparisons (two-sided) were performed.

The basic principle for the interpretation of the numerical results was the assessment of the difference in the concentration of a given parameter in three subsequent analyses. The root mean square precision errors (absolute and percentage, RMSCV and RMSCV%) were calculated for each patient according to 3 steps: (1) calculating the square of individual CV’s, (2) calculating the mean of squared CV’s, and finally, (3) calculating the square root of the mean [9]

The least significant change (LSC) as an absolute value was calculated for groups with a 95% confidence interval by multiplying the absolute root mean square precision error (RMSCV) by a factor of 2.77 [7,8]. The least significant change as a percentage (LSC%) was calculated for groups with a 95% confidence interval by multiplying the percentage root mean square precision error (RMSCV%) by a factor of 2.77 [7,8]. When the calculated LSC value is exceeded, it is assumed with a 95% probability that the change is significant, i.e., the change is greater than the repeatability error of the method and is clinically significant. Spearman correlation tests were performed to analyze the root mean square absolute and percentage precision errors (RMSCV and RMSCV%) in relation to the 25(OH)D concentration values.

## 3. Results

### 3.1. General Characteristics

The average 25(OH)D concentration in the study group was 28.5 ng/mL ± 11.7. The median of 25(OH)D in studied patients was 28.3 ng/mL, with 4.2 ng/mL and 80.8 ng/mL as the minimum and maximum concentrations. In the studied group, 22% of patients showed vitamin D deficiency, and the 25(OH)D concentration values were lower than 20 ng/mL. Suboptimal (generally called insufficient) concentration, i.e., 25(OH)D > 20–30 ng/mL, was reported in 36% of patients, while optimal 25(OH)D concentration values (>30–50 ng/mL) were noted in 38.3% of patients. High concentrations, 25(OH)D > 50–100 ng/mL, were recorded in four patients, representing 2.7% of the group under study.

### 3.2. Calculation of Root Mean Square Precision Error (RMSCV) and Least Significant Change (LSC)

The calculated root mean square precision error, absolute (RMSCV) and percentage (RMSCV%), and the least significant change, absolute (LSC) and percentage (LSC%), for the entire study group and separate subgroups are presented in Table 1, Table 2, Table 3, Table 4 and Table 5.

It was revealed that the absolute value of RMSCV was 1.5 ng/mL, and the absolute LSC calculated in ng/mL, based on triple assessments of 25(OH)D (n = 450), was 4.0 ng/mL (Table 1). The root mean square percentage precision error (RMSCV%) and percentage LSC (LSC%) appeared to be markedly higher, 4.8% and 13.2%, respectively.

### 3.3. Absolute and Percentage Root Mean Square Precision Errors

When root mean square precision errors (RMSCV and RMSCV%) were analyzed in relation to gender, age, and time period of the year, there were no statistically significant differences between girls and boys (Table 2) between different age groups (Table 3) or when a time period of year was compared (Table 4).

### 3.4. Least Significant Change (LSC) in Relation to Age, Gender, Time of Assays, and 25(OH)D Concentrations

There were no statistically significant differences between the calculated LSC values in the studied subgroups according to gender (for absolute LSC, *p* = ns; for percentage LSC, *p* = ns; Table 2) and age (*p* value for absolute LSC, *p* = ns; *p* value for percentage LSC, *p* = ns; Table 3).

When divided according to the time period (date of assay), there were no statistically significant differences between the calculated LSC values in the studied subgroups (*p* = ns for LSC absolute; *p* = ns for percentage LSC; Table 4).

When the 25(OH)D concentration was controlled for, there were no statistically significant differences between the calculated LSC values for the root mean square percentage error (*p* = ns, Table 5); however, there were statistically significant differences between the calculated LSC values for the root mean square absolute error (*p* < 0.0001) in the subgroups under study (Table 6).

The calculated absolute LSC value in the subgroup with vitamin D deficiency, with concentrations of 25(OH)D < 20 ng/mL, showed statistically significant differences from all other subgroups with higher 25(OH)D concentrations (Table 6). The subgroups that did not differ significantly from each other were those with suboptimal concentrations (20–30 ng/mL) and optimal concentrations (30–50 ng/mL). The subgroup with high 25(OH)D concentrations (50–100 ng/mL) differed significantly only from the subgroup with low 25(OH)D concentrations (<20 ng/mL) (Table 6).

Finally, Spearman correlation tests between the 25(OH)D concentration values and the absolute and percentage root mean square precision errors (RMSCV and RMSCV%) were performed, showing the highly significant correlation, with *r* = 0.44 for the absolute root mean square precision error (RMSCV; *p* < 0.0001) but not for the root mean square precision error expressed in percentages (RMSCV%; *r* = −0.14; *p* = 0.08).

## 4. Discussion

In clinical practice, a physician analyzing a single test result considers several aspects. The main issue is whether the measurement is reliable, which is defined as the high accuracy and precision of the test. These qualities should be confirmed by control procedures used by a laboratory.

The interpretation of numerical data appears to be more difficult when it is necessary to compare two test results obtained in a time interval. The correct direction of expected changes is one of the determinants of a patient’s current health condition. A favorable change in the test result can take different directions. When the concentration of a marker of a disease entity is assessed, a significant decrease or increase is expected. In the case of a substance being a component of systemic homeostasis (e.g., vitamin D), an increase in the concentration over a certain period may be a confirmation of the correct supplementation. It may also be desirable to maintain a relatively constant blood concentration over a defined period, e.g., a drug administered intravenously. In all the above cases, being able to determine whether there has been a change or not is the basic question posed by the person assessing the test results. Another question is the extent of the change that should be considered significant for the treatment process. The statistical variability calculated using a standard method does not necessarily translate into clinical significance. In the evaluation of laboratory results, a skilled physician often decides, based on their own experience and intuition, whether a noticeable difference between two results should be considered. This situation is comparable to assessing a patient’s health condition by determining the minimal clinically important difference (MCID) based on the physician’s subjective judgment and the patient’s well-being.

This paper tested for the first time the practical applicability of the least significant change (LSC) for the assessment and interpretation of laboratory results assessing vitamin D supply. To the best of our knowledge, there are no absolute or percentage LSC data for the total 25(OH)D concentrations assessed in children or adults. The concept of the study assumed both intra-individual variability and that resulting from the approximately 10-month analysis period, showing the influence of the period of the year, i.e., possible seasonal variations in the IDS-iSYS (IDS 25 Vit^s^) system [15,16,17] and, most likely, differences in concentrations between the winter and summer months [18,19,20]. It can be assumed that the model of the research can be compared to the routine of daily laboratory work.

Our study was the first to perform complex analyses of the absolute root mean square precision error, relative root mean square precision error, and absolute and relative least significant change (LSC and LSC%) for 25(OH)D assays performed in 450 samples from 150 patients, mainly during the growth and maturation period, using IDS-iSYS system of measurement [File S1]. Our main findings are that absolute and relative root mean square precision error values (RMSCV, RMSCV%) and, consequently, least significant changes (LSC and LSC%—calculated as RMSCV or RMSCV% times 2.77) were not statistically different when age, gender, and the time period of the assessments were controlled for. The potentially expected dependence of the LSC on the patient’s age—despite approximately 2-fold higher absolute LSC values in the group of patients below the age of 1 than in the other groups—was not statistically confirmed (*p* > 0.05). Further, based on the data obtained from the analysis of the 25(OH)D concentration in the selected group of patients, the absolute LSC value was 4.0 ng/mL, whereas the LSC expressed as % appeared as 13.2%; however, both methods were proposed by us as applicable for this specific IDS-iSYS assay system only. In general terms, our findings showed that two or more 25(OH)D concentrations, when compared, will differ significantly from each other in a clinical and diagnostic context when the difference between them is greater than 4.0 ng/mL. Consequently, our study pointed out that the monitoring procedure with the use of the LSC, at least in pediatric cases at our hospital, should be based on the absolute root mean square precision error (RMSCV) but not on the relative/percentage root mean square precision error (RMSCV%), which appeared to be less sufficient.

In contrast, the 25(OH)D concentration appeared to be the only variable that influenced the obtained LSC. In the subgroup with vitamin D deficiency (<20 ng/mL), the obtained LSC value was 2.2 ng/mL (14.7%), which was lower than in the other subgroups (Table 6) and was statistically significantly different. It appeared that in subgroups with a suboptimal (>20–30 ng/mL) and optimal (>30–50 ng/mL) 25(OH)D concentrations, the LSC values were not significantly different from each other; the calculated LSCs were 3.7 ng/mL (14.2%) and 4.1 ng/mL (10.9%), respectively. The last subgroup, with high 25(OH)D concentration values (>50 ng/mL), differed statistically significantly in the LSC value only from the subgroup with vitamin D deficiency (<20 ng/mL). However, the comparison of that subgroup to the others was difficult due to its small size (n = 4). It was also observed that the absolute root mean square CV value and that of the absolute LSC when analyzed according to increasing 25(OH)D concentrations (Table 5) tended to increase, which was not observed when comparing the relative/percentage values of these variables. But Spearman correlation tests between the 25(OH)D concentration values and the absolute and percentage root mean square precision errors (RMSCV and RMSCV%) provided an answer—the results showed a significant correlation with the absolute root mean square precision error (RMSCV; *p* < 0.0001) but not with the root mean square precision error expressed in percentages (RMSCV%). Further, the correlation between 25(OH)D and the root mean square CV% tended to be negative.

The main limitation of our study is that the impact of lot-to-lot variations (LTLVs) in IDS 25 VitD kits (IDS 25 Vit^s^) was not investigated. Lot-to-lot variations might negatively affect assay accuracy, precision, and specificity, leading to some probable uncertainty in the reported results. These variations are considered the main problem for immunoassays. Consequently, to estimate the LSC while considering LTLVs, three aliquots of the serum sample should be measured by three different lots of IDS 25 VitD kits. However, the basic assumption of the study was to perform the tests in the model of everyday, routine laboratory work. Our laboratory had the ability to work on a currently available series of kits from the manufacturer (the so-called LOT number). The manufacturer does not provide us with batches of kits that have already been withdrawn or are planned to be released, so they are not used for everyday work. Studies comparing batches of kits at different times are probably performed only in the internal structures of a given manufacturer (in this case, IDS). It should be highlighted that this study is the first study providing LSC values for 25(OH)D assessments in pediatric patients, which are typical cases in our hospital. This study was not constructed with a perfect design; therefore, future studies should include lot-to-lot variations for analysis. The authors of the study believe that the LSC values presented above allow for their practical use in the interpretation and assessment of 25(OH)D concentration results, considering the discussed limitations. It is advisable to extend the study to a larger patient population, include lot-to-lot variations in analyses, and perform such studies for other immunoassay manufacturers and diagnostic laboratories. Finally, the expected study population should consist of a comparable number of participants with different 25(OH)D concentrations.

The main finding of the study is the obtained absolute LSC value, which, for the purpose of interpretation of the results, can be assumed to be approximately 4.0 ng/mL. Thus, a person comparing two or more 25(OH)D concentrations of the same patient obtained at different periods of time can state with a probability of 95% that, when the values differ by more than 4.0 ng/mL, the difference is of diagnostic significance. This diagnostic assumption can be made without reservations for suboptimal and optimal concentrations, i.e., 25(OH)D > 20 ng/mL up to 50 ng/mL, which is the case for over 75% of the patients in the studied group. The situation appeared slightly different for low 25(OH)D concentrations (<20 ng/mL), where the absolute LSC concentration appeared to be 2.2 ng/mL. In turn, in the high-concentration range (>50 ng/mL), the value of the calculated absolute LSC turned out to be several times greater, 11.8 ng/mL. However, the small size of this group (n = 4) prevented an effective comparison with the others and the diagnostic use of this LSC.

## 5. Conclusions 

The practical conclusion of our study, which should be considered for medical doctors (MDs) dealing with results of 25(OH)D concentrations after their medical intervention, is the LSC value of 4 ng/mL. This LSC value, which was calculated and presented for the first time in this study, should be considered an additional tool for the diagnosis of the effects of vitamin D supplementation. MDs should use the LSC value when comparing 25(OH)D test results simultaneously with other data concerning the patient (medical history, results of other laboratory tests, dosing of vitamin D prescribed for a patient, imaging tests, etc.). The LSC should be considered among other clinical issues by MDs. The patient and his/her health are the most important for MDs.

## Figures and Tables

**Table 1:** Root mean square precision error (RMSCV and RMSCV%) and least significant change (LSC and LSC%) across the study group of 150 patients with triple assessments of 25(OH)D.

Variable	Number of Patients	Number of Samples	Calculated Value
RMSCV-absolute [ng/mL]	150	450	1.5 ng/mL
RMSCV%-percentage [%]	150	450	4.8%
LSC-absolute [ng/mL]	150	450	4.0 ng/mL
LSC%-percentage [%]	150	450	13.2%

**Table 2:** Root mean square precision error (RMSCV and RMSCV%) and least significant change (LSC and LSC%) in relation to gender.

Variable	Gender	Number of Patients	Number of Samples	Calculated Value
RMSCV [ng/mL]	F	67	201	1.6 ng/mL
RMSCV% [%]				4.7%
LSC [ng/mL]				4.4 ng/mL
LSC [%]				13.0%
RMSCV [ng/mL]	M	83	249	1.4 ng/mL
RMSCV% [%]				4.8%
LSC [ng/mL]				3.8 ng/mL
LSC% [%]				13.4%

**Table 3:** Root mean square precision error (RMSCV and RMSCV%) and least significant change (LSC and LSC%) in relation to age.

Variable	Age [Years Old]	Number of Patients	Number of Samples	Calculated Value
RMSCV [ng/mL]	<1	17	51	2.5 ng/mL
RMSCV% [%]				4.9%
LSC [ng/mL]				6.9 ng/mL
LSC% [%]				13.5%
RMSCV [ng/mL]	1–3	15	45	1.6 ng/mL
RMSCV% [%]				4.9%
LSC [ng/mL]				4.4 ng/mL
LSC% [%]				15.4%
RMSCV [ng/mL]	>3–12	43	129	1.1 ng/mL
RMSCV% [%]				4.4%
LSC [ng/mL]				3.2 ng/mL
LSC% [%]				12.2%
RMSCV [ng/mL]	>12–18	64	192	1.2 ng/ml
RMSCV% [%]				4.7%
LSC [ng/mL]				3.4 ng/mL
LSC% [%]				12.9%
RMSCV [ng/mL]	>18	11	33	1.5 ng/mL
RMSCV% [%]				5.4%
LSC [ng/mL]				4.1 ng/mL
LSC% [%]				14.9%

**Table 4:** Root mean square precision error (RMSCV and RMSCV%) and least significant change (LSC and LSC%) in relation to the time period of a year.

Variable	Time Period	Number of Patients	Number of Samples	Calculated Value
RMSCV [ng/mL]	Group I (Winter set)	30	90	1.1 ng/mL
RMSCV% [%]				4.0%
LSC [ng/mL]				3.1 ng/mL
LSC% [%]				11.0%
RMSCV [ng/mL]	Group II (late Winter-Spring set)	30	90	1.2 ng/mL
RMSCV% [%]				4.8%
LSC [ng/mL]				3.2 ng/mL
LSC% [%]				13.2%
RMSCV [ng/mL]	Group III (Spring set)	30	90	2.0 ng/mL
RMSCV% [%]				5.8%
LSC [ng/mL]				5.5 ng/mL
LSC% [%]				16.0%
RMSCV [ng/mL]	Group IV (late Spring-Summer set)	30	90	1.4 ng/mL
RMSCV% [%]				4.7%
LSC [ng/mL]				4.0 ng/mL
LSC% [%]				13.1%
RMSCV [ng/mL]	Group V (late Summer-Fall set)	30	90	1.4 ng/mL
RMSCV% [%]				4.4%
LSC [ng/mL]				3.9 ng/mL
LSC% [%]				12.3%

**Table 5:** Root mean square precision error (RMSCV and RMSDCV%) and least significant change (LSC and LSC%) in relation to 25(OH)D concentration.

Variable	25(OH)D Concentration [ng/mL]	Number of Patients	Number of Samples	Calculated Value
RMSCV [ng/mL]	<20	33	99	0.8 ng/mL
RMSCV% [%]				5.3%
LSC [ng/mL]				2.2 ng/mL
LSC% [%]				14.7%
RMSCV [ng/mL]	>20–30	53	159	1.3 ng/mL
RMSCV% [%]				5.1%
LSC [ng/mL]				3.7 ng/mL
LSC% [%]				14.2%
RMSCV [ng/mL]	>30–50	60	180	1.5 ng/mL
RMSCV% [%]				3.9%
LSC [ng/mL]				4.1 ng/mL
LSC% [%]				10.9%
RMSCV [ng/mL]	>50–100	4	12	4.3 ng/mL
RMSCV% [%]				6.1%
LSC [ng/mL]				11.8 ng/mL
LSC% [%]				16.9%

**Table 6:** Absolute least significant change (LSC) in relation to 25(OH)D concentration. *p* value for multiple (two-sided) comparisons—Kruskal–Walli’s test. Independent variable: 25(OH)D concentration.

25(OH)D Concentration [ng/mL]	<20	20–30	>30–50	>50–100
<20	XXX	<0.05	<0.0001	<0.005
20–30	<0.05	XXX	ns	ns
>30–50	<0.0001	ns	XXX	ns
>50–100	<0.005	ns	ns	XXX

ns, not significant; XXX, not applicable for statistical analysis.

## Data Availability

The data presented in this study are available on request from the corresponding author. The data are not publicly available due to privacy, legal and ethical reasons.

## Supplementary Materials

The following supporting information can be downloaded at: https://www.mdpi.com/article/10.3390/nu17132246/s1, File S1: Baseline data of 25(OH)D concentrations.
